# Cyclodextrin-Induced Suppression of the Crystallization of Low-Molar-Mass Poly(ethylene glycol)

**DOI:** 10.1021/acspolymersau.4c00024

**Published:** 2024-05-02

**Authors:** Ian W. Hamley, Valeria Castelletto

**Affiliations:** School of Chemistry, Food Biosciences and Pharmacy, University of Reading, Whiteknights, Reading RG6 6AD, U.K.

**Keywords:** crystallization, cyclodextrins, rotaxanes, SAXS/WAXS, poly(ethylene glycol), DSC

## Abstract

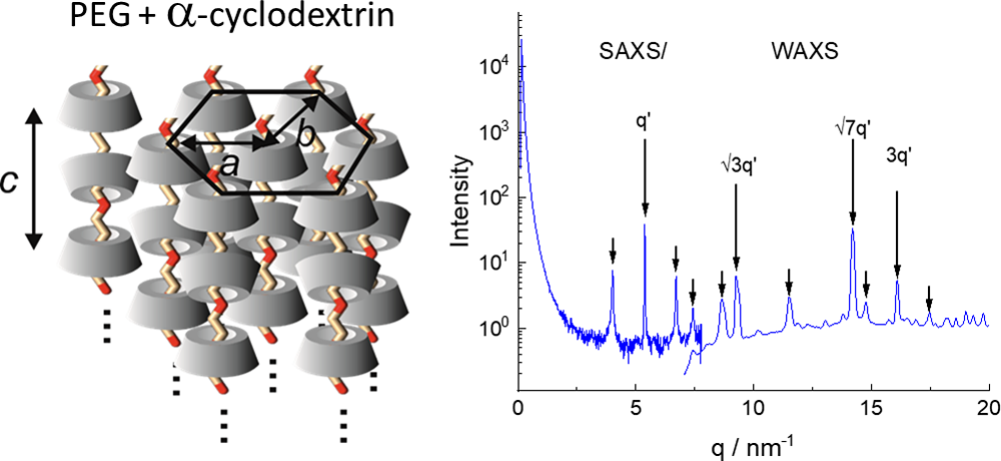

We examine the effect of alpha-cyclodextrin (αCD) on the crystallization of poly(ethylene glycol) (PEG) [poly(ethylene oxide), PEO] in low-molar-mass polymers, with *M*_w_ = 1000, 3000, or 6000 g mol^–1^. Differential scanning calorimetry (DSC) and simultaneous synchrotron small-/wide-angle X-ray scattering (SAXS/WAXS) show that crystallization of PEG is suppressed by αCD, provided that the cyclodextrin content is sufficient. The PEG crystal structure is replaced by a hexagonal mesophase of αCD-threaded polymer chains. The αCD threading reduces the conformational flexibility of PEG and, hence, suppresses crystallization. These findings point to the use of cyclodextrin additives as a powerful means to tune the crystallization of PEG (PEO), which, in turn, will impact bulk properties including biodegradability.

## Introduction

Control of self-assembly through judicious use of noncovalent interactions is an important theme in contemporary materials chemistry research.^1^ Cyclodextrins are cyclic oligosaccharides with 6, 7, and 8 linked sugars in αCD, βCD, and γCD, respectively, that form inclusion complexes with polymers due to threading of the rotaxane molecules on the polymer chains.^2^ Cyclodextrins are generally inexpensive, plant-derived compounds (obtained from starch) of interest as additives in the development of renewable materials such as metal–organic frameworks (MOFs).^3−5^ They can be modified with hydrophobic substituents to enable complexation with hydrophobic molecules and such derivatives are used for example to extract cholesterol from cells.^6−8^ Cyclodextrins have been widely explored for applications in pharmaceutics since they can be used to create water-soluble complexes with hydrophobic drugs.^9−14^ Many other potential uses arising from host–guest interactions have been demonstrated based on supramolecular polymer or amphiphile formation.^12,15−20^ Among cyclodextrins, αCD contains six glucose-derived saccharides and contains a small cavity, which can thread around polymers including polyethylene glycol (PEG),^21−24^ due to hydrogen bonding interactions with the ether oxygen atoms.

Cyclodextrins have been investigated as additives to tune polymer crystallization. The addition of αCD to polymers can enhance crystallization from the melt, i.e., the αCD acts as a nucleating agent,^25^ due to the formation of inclusion complexes. This has been reported for poly(3-hydroxybutyrate).^26,27^ The formation of inclusion complexes of αCD has also been reported for polymers including polyesters such as poly(ε-caprolactone) [PCL],^28^ polyethers such as poly(ethylene glycol) PEG [i.e., hydroxyl-terminated poly(ethylene oxide), PEO]^21,29,30^ and block copolymers such as oligomeric copolymers containing PEO^31^ and/or PCL.^32,33^ Inclusion complexes are formed by polyolefins such as poly(isobutylene) with βCD and γCD.^34,35^ Other studies on the inclusion complex formation of polymers with cyclodextrins have been reviewed.^33^ Addition of inclusion complexes (not just the αCD itself) accelerates the nucleation and crystallization of several polymers including PCL, poly(butylene succinate), and PEG (*M*_w_ = 20,000 g mol^–1^).^36,37^ In contrast, αCD forms inclusion complexes with aliphatic polyesters poly(3-hydroxypropionate), poly(4-hydroxybutyrate), and PCL which leads to suppression of crystallization, as revealed by DSC and wide-angle XRD.^38^ However, to date, the effect on the crystallization of polyethers such as PEG of αCD itself (not preformed inclusion complexes) has not been examined. Since the enzymatic degradation of polymers can be enhanced by reducing crystallinity,^39−44^ methods to suppress crystallization resulting from the complexation of certain polymers with specific cyclodextrins, can be used to improve polymer biodegradation,^33,45^ a very important societal challenge.

Here, we report on an investigation of the influence of αCD on the crystallization behavior of low-molar-mass PEG (three molar masses). DSC and simultaneous SAXS/WAXS were used to investigate the behavior of dry/melt samples, and heat/cool experiments revealed the unexpected suppression of PEG crystallization in complexes with sufficiently high αCD content. Instead, the PEG/αCD complexes form a hexagonal structure, as revealed by analysis of combined SAXS/WAXS data over an extended *q* range. These observations are rationalized based on the effect of αCD threaded onto PEG chains in restricting the conformational flexibility of the polymer.

## Experimental Section

### Materials and Sample Preparation

Samples of PEG1000, PEG3000, PEG6000, and α-cyclodextrin (αCD) were obtained from Sigma-Aldrich (U.K.) and are USP reference grade products. PEG1000 has stated molar mass *M*_w_ = 950–1050 g mol^–1^, while for PEG3000 *M*_w_ = 2959 g mol^–1^, for PEG6000, it is quoted as *M*_w_ = 6000 g mol^–1^.

Table S1 lists wt % concentrations and molar ratios for the samples studied in this work.

The wt % PEG in binary samples was calculated according to wt % PEG = 100 × [weight_PEG_/(weight_aCD_ + weight_PEG_ + weight_water_)]. The corresponding molar concentration of PEG was calculated using only weight_PEG_ and the volume of water equivalent to that of weight_water_. An equivalent method was used to calculate the wt % and molar concentration of αCD in binary samples (Table S1).

To prepare the samples, convenient weighed amounts of αCD and water were placed in a vial. The αCD was dissolved using ultrasound and vortexing for 10 min. The transparent αCD solution was then used to dissolve a convenient weighed amount of PEG. As with the previous step, the PEG was dissolved using ultrasound and vortexing for another 10 min. The resulting αCD/PEG solution was allowed to rest for 24 h and then dried on a glass slide for 24 h. The dried powder was recovered from the glass slide by scratching with a scalpel. Samples were then stored under vacuum before being studied by X-ray scattering or DSC.

### Differential Scanning Calorimetry (DSC)

Experiments were performed by using a TA-Q200 DSC instrument. Samples were prepared as detailed in the Materials and Sample Preparation section, and the resulting powder was loaded into sealed DSC pans. Temperature ramps were performed in the range 19 °C → −40 °C → 120 °C → −40 °C with a cool/heat rate of 10 °C/min.

### Simultaneous Small-Angle/Wide-Angle X-ray Scattering (SAXS/WAXS)

Simultaneous SAXS/WAXS experiments were carried out at DUBBLE (BM26)^46^ at the ESRF (Grenoble, France) using an X-ray beam with a wavelength of 12 keV. Samples were prepared as detailed in the Materials and Sample Preparation section, and the resulting powder was loaded in sealed DSC pans with mica windows.

The WAXS signal was acquired with a Pilatus 300 K–W (1472 × 195 pixels) detector that is characterized by a pixel size of 172 μm × 172 μm, while the SAXS signal was recorded with a Pilatus 1 M with a detector size (981 × 1043) with a pixel size of 172 μm × 172 μm at a sample to detector distance of ca. 1.45 m. Alumina (α-Al_2_O_3_) was employed to calibrate the wavenumber (*q* = 4πsin*q*/λ) scale for the WAXS and AgBe for the SAXS scale. The SAXS data was corrected for the background of an empty DSC pan. Both SAXS and WAXS data were corrected for transmission before being integrated into 1D intensity profiles using the software Bubble^47^ and both are expressed in arbitrary units.

## Results

**Figure 1:**
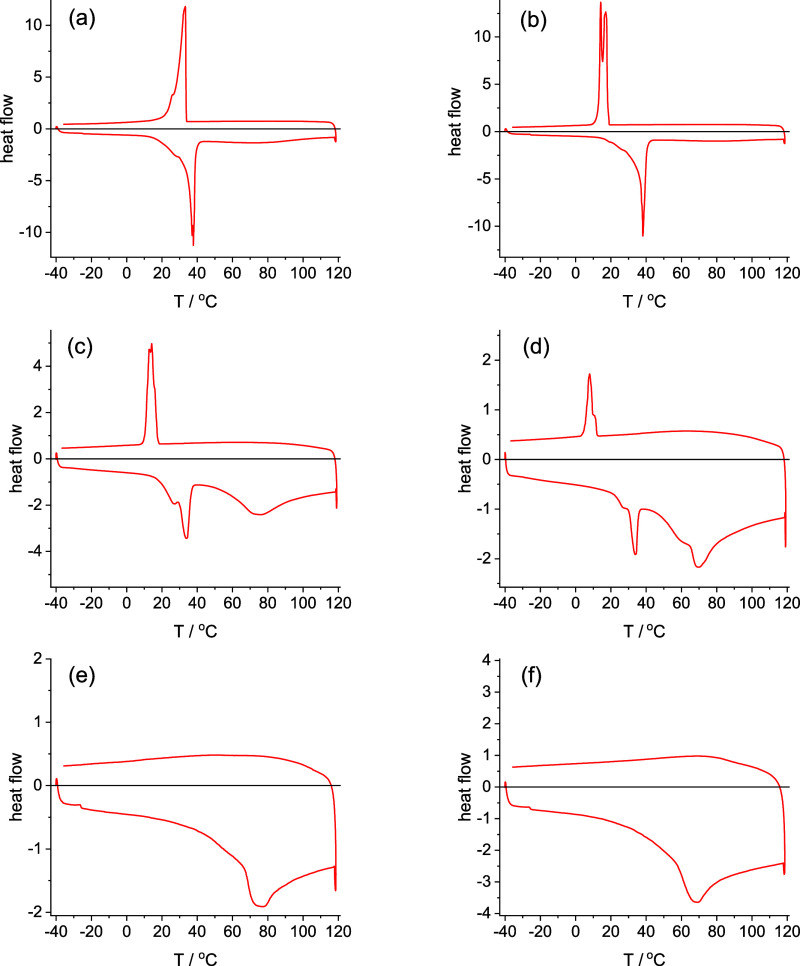
DSC data (endo down) measured for PEG1000 with (a) 0, (b) 0.03 wt %, (c) 1.6 wt %, (d) 3.6 wt %, (e) 8 wt %, and (f) 13 wt % αCD. The first heating ramp is from −40 to 120 °C and the second cooling ramp is from 120 to −40 °C.

Differential scanning calorimetry (DSC) was first used to identify phase transitions associated with PEG melting and crystallization and the influence of α-cyclodextrin (αCD) on this in blends with varying αCD content. Figure 1 shows DSC thermograms obtained for PEG1000 and blends with varying αCD content. The data for PEG1000 alone in Figure 1a show a melting endotherm with a peak at *T*_m_ = 37.8 °C and a crystallization exotherm maximum at *T*_c_ = 33.2 °C for the second cooling ramp. Upon incorporation of 0.03 wt % αCD, the melting endotherm (Table 1) and crystallization exotherm are retained (Figure 1b), although in the latter case, there is greater undercooling (hysteresis) than for the PEG1000, and there is evidence for fractionated crystallization. The melting/crystallization peaks are greatly reduced but still present in the complexes with 1.6 and 3.6 wt % αCD (Figure 1c,d). Crystallization occurs with peaks at *T*_c_ = 14.0 °C (1.6 wt % αCD) and *T*_c_ = 7.9 °C (3.6 wt % αCD). In contrast to these results, there is no evidence for PEG melting/crystallization peaks in the DSC data (Figure 1d,e) for samples containing 8 or 13 wt % αCD which, as for αCD itself (SI Figure S1), just show a broad peak on heating due to water loss, starting at 70 °C. DSC data for PEG3000 and blends with αCD are shown in SI Figure S2, and for PEG6000 and blends with αCD in SI Figure S3. For PEG3000, the DSC data show that crystallization is suppressed in the blend with 10 wt % αCD and is almost absent in the blend with 5 wt % αCD, and for PEG6000, there is no recrystallization exotherm for samples with 7 wt % αCD or more (and it is only weakly present for the 5 wt % αCD blend). The values of melting temperature (*T*_m_), crystallization temperature (*T*_c_), and melting enthalpy Δ*H*_m_ for all studied blends (and the polymers without αCD) are listed in Table 1 which shows the general trend for a given PEG molar mass for *T*_m_, *T*_c_, and Δ*H*_m_ to all reduce upon addition of αCD until melting (crystallization) is completely suppressed. The DSC data for all three PEG samples thus indicate that PEG crystallization is suppressed in blends containing sufficient αCD.

**Table 1:** Melting and Crystallization Temperatures and Melting Enthalpy Values from DSC Data in Figures 1, S2, and S3

sample	*T*_m_ (°C) (peak)	*T*_c_ (°C) (onset)	Δ*H*_m_ (J g^–1^)
PEG1000	37.8	33.7	145.4
PEG1000 + 0.03 wt % αCD	38.1	18.0	140.8
PEG1000 + 1.6 wt % αCD	33.8	16.3	27.8
PEG1000 + 3.6 wt % αCD	33.8	10.0	15.6
PEG3000	57.9	38.0	165.1
PEG3000 + 0.1 wt % αCD	57.3	28.8	170.3
PEG3000 + 5 wt % αCD	55.3	22.9	9.9
PEG6000	60.6	41.8	183.3
PEG6000 + 0.2 wt % αCD	59.5	35.4	140.3
PEG6000 + 5 wt % αCD	51.9	21.0	7.3

**Figure 2:**
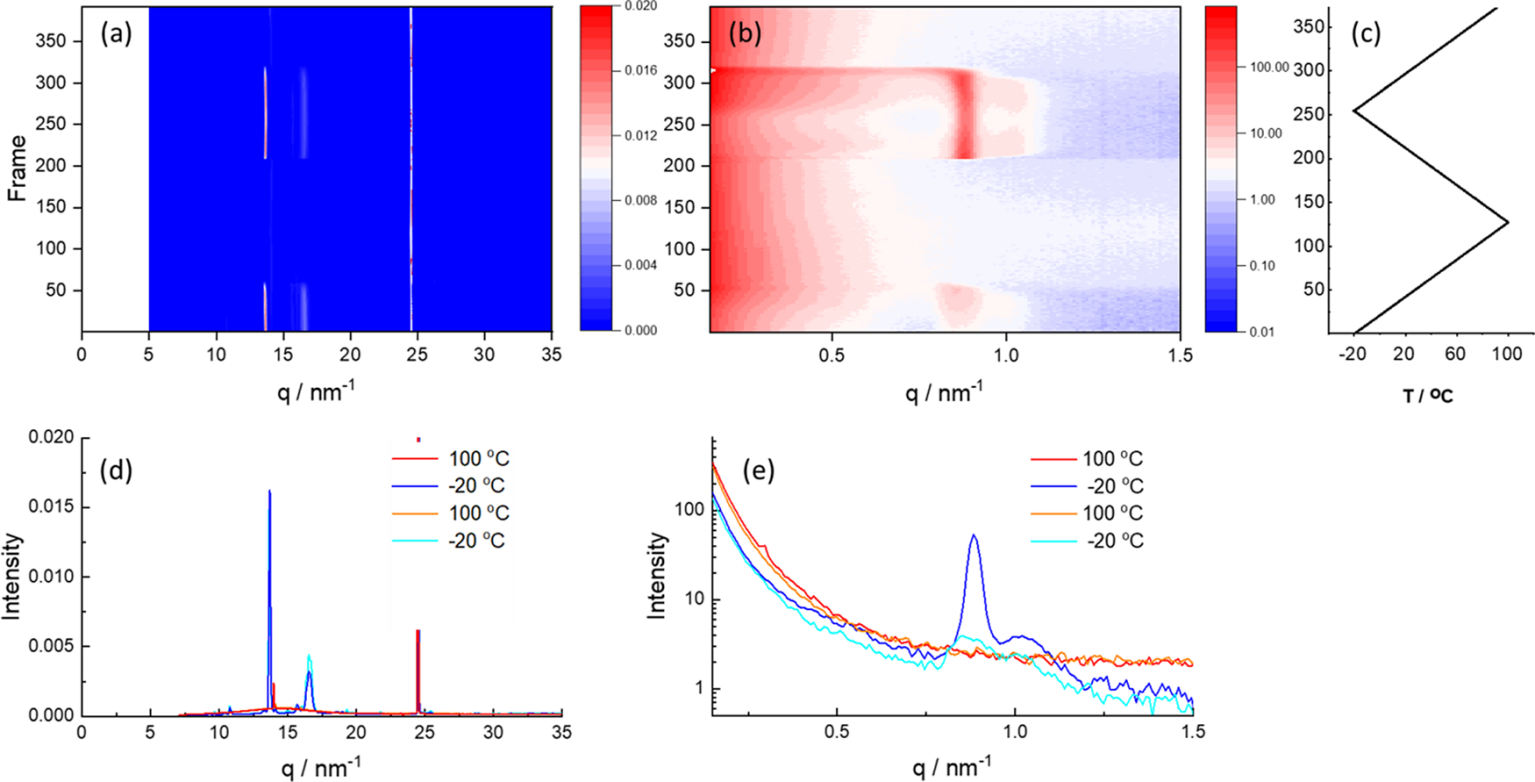
SAXS/WAXS data for PEG1000 during a heat/cool/heat cycle at 5 °C/min (a) WAXS data heatmap (intensity for each frame stacked vertically), (b) SAXS data heatmap, (c) temp ramp profile corresponding to the heatmaps in (a, b), (d) selected frames of WAXS data at the temperatures indicated—cyan: −20 °C (start), orange: 100 °C (first heat), blue −20 °C (second cool), and red 100 °C (second heat) (the peak near *q* = 24 nm^–1^ is due to a reflection from the mica window), and (e) selected frames of SAXS data (same color scheme as for WAXS).

The versatile method of simultaneous synchrotron SAXS/WAXS^48^ was used to examine structural features of ordering including PEG crystallization in the PEG/αCD blends across length scales associated with superstructure formation (SAXS) and local ordering extending down to the atomic level (WAXS). We first consider measurements for PEG1000 before briefly discussing data for PEG3000 and PEG6000 which present features similar to those for the lowest molar mass PEG studied. The WAXS data for PEG1000 (Figure 2a) show the disappearance of PEG crystal reflections on heating and reversible reappearance on cooling, and remelting on second heating (the temperature profile is shown in Figure 2c). The WAXS data for the crystalline PEG (Figure 2d) were indexed using the published unit cell data for PEG (SI Figure S4).^49,50^ The SAXS data in Figure 2b show reversible changes in the scattering peak centered at q* = 0.89 nm^–1^ (Figure 2e), which is due to the formation of PEG lamellae upon crystallization. There is also a weak broad shoulder peak centered at *q* = 1.03 nm^–1^ due to a secondary population of crystalline lamellae. The principal peak is accompanied by higher-order reflections at 2q* and 3q* (SI Figure S5) confirming a lamellar structure with a spacing *d* = 7.06 nm. This is comparable to the estimated length of PEG1000 in an extended conformation, approximated as *l*_PEG_/nm = 0.095 *z*_E_, where *z*_E_ is the number of chain atoms (C and O),^51^ which for PEG1000 gives *l*_PEG_ = 6.47 nm. The slight discrepancy is ascribed to an underestimated average degree of polymerization in the sample. The observed *d*-spacing implies that PEG crystallizes as an extended chain crystal.

**Figure 3:**
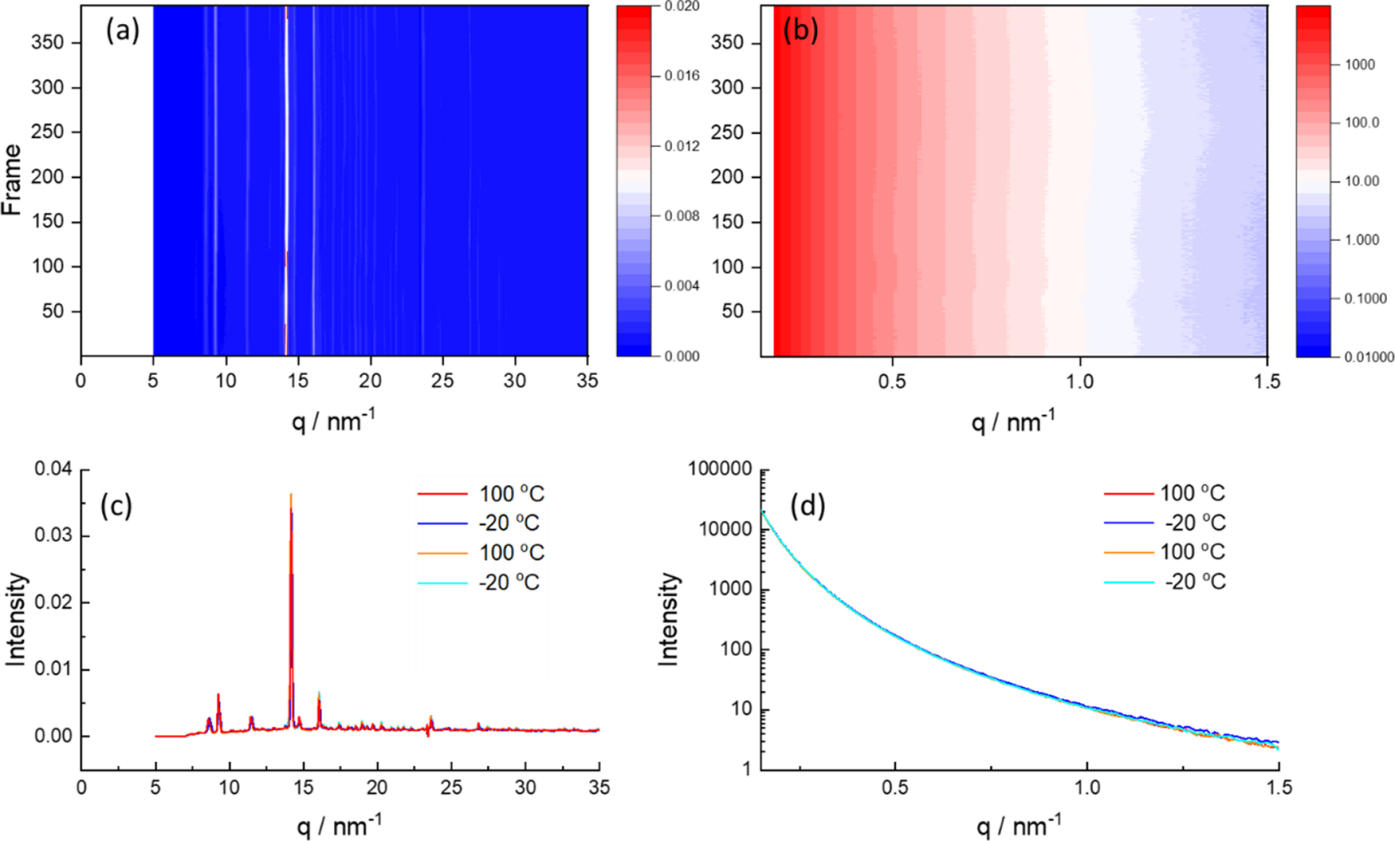
SAXS/WAXS data for PEG1000 + 8 wt % αCD during a heat/cool/heat cycle at 5 °C/min (a) WAXS data heatmap (intensity for each frame stacked vertically), (b) SAXS data heatmap, (c) selected frames of WAXS data at the temperatures indicated—cyan: −20 °C (start), orange: 100 °C (first heat), blue −20 °C (second cool), red 100 °C (second heat), (d) selected frames of SAXS data (same color scheme as for WAXS).

We next consider the SAXS/WAXS data for PEG1000 in the blends with αCD. Similar behavior was observed to that observed for PEG1000 alone; i.e., WAXS peaks for crystalline PEG and reversible melting/crystallization were noted for a blend with low αCD content (0.003 wt %) as shown in SI Figure S6. In this and subsequent plots of SAXS/WAXS data, the temperature ramp profiles are omitted since they are the same as those in Figure 2c (except for PEG3000, where the maximum temperature was 120 °C to check for any possible higher temperature melting, which was not observed). However, a very distinct behavior was noted for blends with high αCD content for which DSC indicated the suppression of PEG crystallization. Figure 3 shows simultaneous SAXS/WAXS data for PEG1000 with 8 wt % αCD. The WAXS data in Figure 3a,c show the absence of reflections due to PEG crystallization, and no significant temperature dependence is observed across the heat–cool cycles. Importantly, the WAXS data corresponds to neither that of αCD alone nor PEG1000 (SI Figure S7). These points to the formation of a distinct αCD-PEG inclusion complex structure, to be discussed shortly. The SAXS data in Figure 3b,d show the absence of features from PEG crystal lamellae, and no temperature dependence, further confirming that the addition of αCD has suppressed PEG crystallization. Blends containing intermediate αCD content (1.6 or 3.6 wt %) show features of PEG crystallization in the SAXS/WAXS data shown in SI Figures S8 and S9 (especially in the WAXS data) although new WAXS peaks arise, in particular sharp temperature-independent peaks including a primary peak at *q* = 14.2 nm^–1^ (*d* = 0.44 nm). These peaks are due to the formation of inclusion complexes with αCD (to be discussed in detail below). SAXS/WAXS data for PEG3000 and blends with αCD shown in SI Figures S10–S13 show similar features, i.e., WAXS peaks from PEG crystals for pure PEG3000 and the blend with αCD = 0.1 wt %, whereas the high αCD content blends (αCD = 5 or 10 wt %) show WAXS patterns dominated by peaks arising from αCD inclusion complex formation (for the 5 wt % blend) or exclusively these features (for the 10 wt % blend). The SAXS data for PEG3000 and blends does not show well-defined peaks from crystal lamellae, although there are some temperature-dependent broad features evident in the heat map WAXS data in SI Figures S10c and S11c.

For PEG6000 (and blends of this polymer with low αCD content), the WAXS data in SI Figures S14a,c and S15a,c show the features of PEG melting and crystallization similar to that observed for PEG1000 and PEG3000. The WAXS data shown in SI Figures S16a,c and S17a show that PEG crystal peaks are suppressed largely or entirely in the blends with 5 or 7 wt % αCD, respectively, consistent with the DSC data in SI Figure S3. For this polymer and the αCD = 0.2 wt % blend, the SAXS data shown in SI Figures S14d and S15d show for the low temperature crystal phase a broad peak centered at *q* = 0.47 nm^–1^ (*d* = 13.4 nm) which may be compared to reported crystal lamellar spacings *d* = 19.6 nm and *d* = 39.8 nm for PEO6000 dimethyl ether, corresponding to once folded or unfolded extended PEO chains.^52^ The observed peak in our data is most likely the third-order reflection from unfolded PEO6000 lamellae. This peak is observed to reversibly melt on heating (SI Figures S14b and S15b).

**Figure 4:**
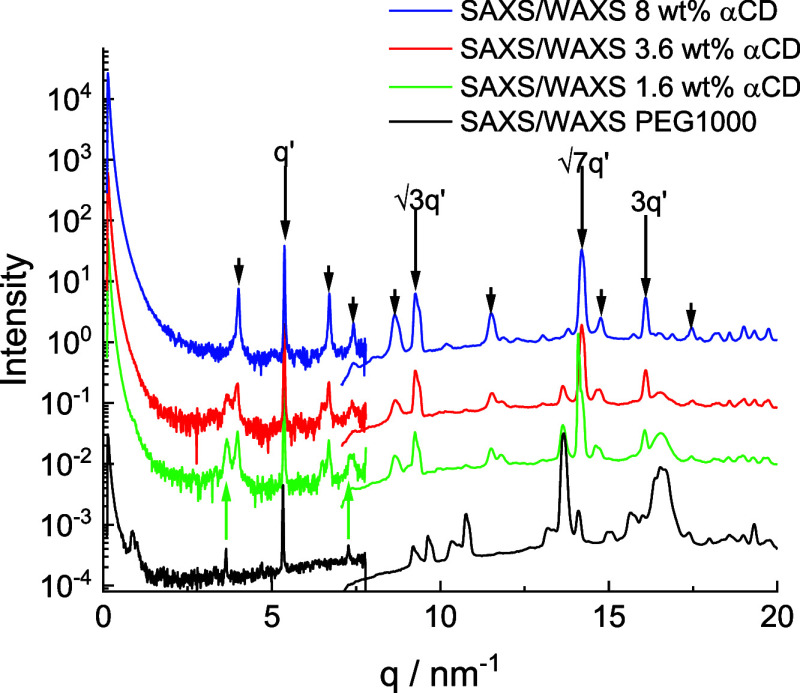
Combined SAXS/WAXS data (at −20 °C, first cooling) for PEG1000 mixtures with αCD content as indicated and PEG1000 alone for comparison. The WAXS data intensity has been scaled to be at approximately the same level of that of the SAXS data, and data is offset for ease of visualization. Peaks due to PEG/αCD complex formation are indicated in black, with the main hexagonal lattice peaks indexed with q′ notation. Peaks due to PEG crystallization are highlighted with green arrows.

The SAXS/WAXS data for all three samples thus show features consistent with the DSC data, i.e., the suppression of PEG crystallization in blends with sufficiently high αCD content. The combination of SAXS/WAXS data in fact provides unique information on the formation of inclusion complexes, and this is now analyzed. SAXS/WAXS data are plotted together for PEG1000 mixtures with high αCD content in Figure 4. Similar features were observed for high αCD content PEG3000 and PEG6000 mixtures (SI Figures S18 and S19). At lower αCD content, some signature peaks from PEG crystallization were retained, as indicated in SI Figure S19.

**Figure 5:**
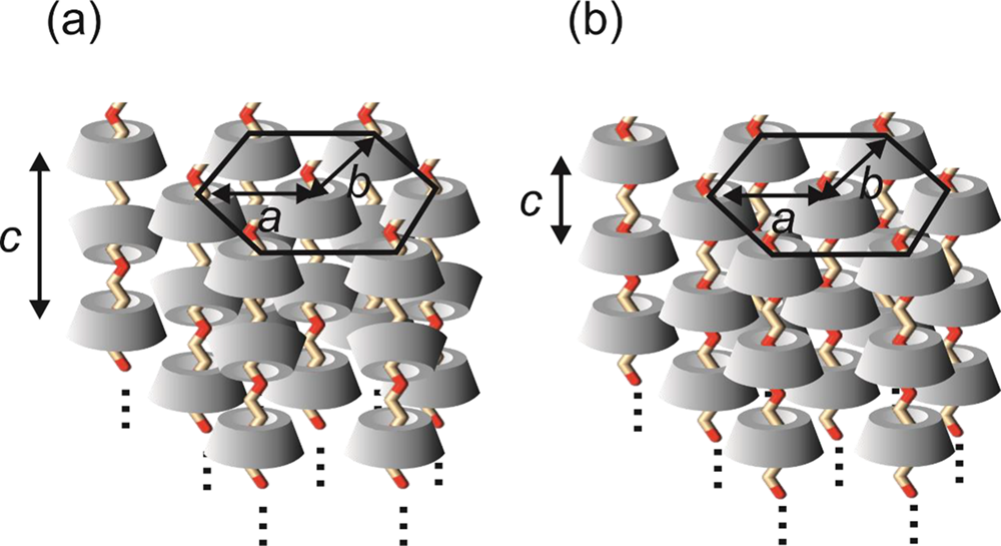
Scheme showing αCD wrapping PEG in complexes, forming a hexagonal lattice (not to scale). (a) Predominant head-to-head (dimer) stacking and (b) Minor head-to-tail stacking.

The SAXS data at high *q* reveal peaks for the blends containing high αCD content, in particular, there is a sharp peak at *q* = 5.37 nm^–1^, marked as q′ in Figures 4, S18, and S19. The peak is absent for αCD alone (Figure S20), therefore it is due to the complexation of αCD with PEG. The combination of SAXS and WAXS in fact provides unique insight into the noncrystalline ordering in the complexes at high αCD content. The stronger peaks in the data in Figures 4, S18, and S19 can be indexed to a hexagonal lattice structure with reflections at *q*′, q*, *q*′, and 3 *q*′. The *q*′ peak at *q* = 14.2 nm^–1^ is enhanced because the corresponding *d*-spacing (*d* = 0.44 nm) is close to the αCD inner diameter.^53^ The expected hexagonal lattice reflection at 2*q*′ is absent due to the degeneracy in hexagonal lattice orientation (0 and 30° rotation). The hexagonal lattice parameter from these reflections is *a* = 1.35 nm. The additional broader set of peaks present in the data in Figures 4, S18, and S19 arise from the ordering out of the plane of the hexagonal lattice including peaks arising from the spacing of the cyclodextrin rings along the *c* axis of the unit cell. The reflections were indexed (SI Table S2) based on a pseudohexagonal lattice with *a* = *b* = 1.31 nm, *c* = 1.51 nm, and an angle γ* = 116° slightly distorted from hexagonal (γ* = 120°). The hexagonal lattice parameters differ slightly from those based on analysis of the stronger hexagonal lattice peaks only (which yielded *a* = 1.35 nm) when accounting for the other broader peaks in a least-squares indexation of the observed peak positions. In fact this indexation is complicated by the probable presence of mixed order due to two possible stackings of the αCD molecules:^29^ head-to-tail or head-to-head (i.e., ordering into dimers) of which the latter is predominant, since it gives rise to a spacing approximately twice the height of an αCD molecule (0.79 nm),^53^ which is close to the length of the *c* axis of the indexed unit cell. The data in Figure 4 show the coexistence of SAXS peaks for the lower two αCD content blends arising from the hexagonal and crystalline structures. This was not observed for the PEG3000 and PEG6000 blends (SI Figures S18 and S19). It indicates a less strong propensity for hexagonal phase formation in the PEG1000 blends with low αCD content. The structure deduced from the SAXS/WAXS data for the PEG/high αCD blends is sketched in Figure 5 which shows the two stacking modes.

## Discussion and Conclusions

Complex formation between PEG1000 and αCD leading to a channel-like crystal structure of αCD-threaded PEG chains was reported in 1990,^21^ although no detailed structure analysis was performed. Here a detailed analysis of the influence of αCD on the crystallization of low-molar-mass PEG (PEG1000, PEG3000, and PEG6000) is provided, and the suppression of PEG crystallization at sufficient αCD loading is demonstrated, which is due to threading of cyclodextrin molecules on the polymer chains. This is shown to lead to a structure comprising a hexagonal array of PEG chains bearing αCD. The hexagonal lattice parameters are similar to those previously reported for a PEG1500/αCD blend based on XRD, although we did not find a notable peak corresponding to *d* = 0.743 nm discussed by Topchieva et al.,^29^ which they assign to the head-to-tail stacking (nondimers) of αCD; however, a minor peak with *d* = 0.724 was recorded, which can be indexed based on the hexagonal unit cell (SI Table S2).^29^ The threading of αCD presumably leads to greatly restricted conformational freedom of the polymer, thus preventing PEG from adopting the extended helical structure characteristic of the crystal state,^49^ but instead the αCD-threaded PEG forms a hexagonal structure with additional ordering of the αCD along the *c* axis of the unit cell.

In contrast to our findings showing suppression of polymer crystallization at high αCD content, it has previously been reported that addition of nonstoichiometric amounts of αCD to polymers, which causes complex formation, can enhance crystallization from the melt (i.e., the αCD acts as nucleating agent),^25^ as exemplified by reports on poly(3-hydroxybutyrate)^26,27^ and on inclusion complexes of αCD with poly(ε-caprolactone), poly(butylene succinate) and PEG (*M*_w_ = 20,000 g mol^–1^).^36,37^ In low molar mass PEG it seems that αCD does not act as a nucleating agent; instead, we propose that it hinders conformational rearrangements of PEG chains preventing crystallization.

For the PEG polymers themselves or low αCD content blends, PEG crystallization was observed. The crystal lamellar *d*-spacing for PEG1000 measured here (*d* = 7.06 nm) is in excellent agreement with that previously reported for linear poly(oxyethylene) dimethyl ether with molar mass 1000 g mol^–1^, *d* = 7.0 nm.^54^ For comparison to PEG6000, Cooke et al. reported crystal lamellar spacings *d* = 19.6 and *d* = 39.8 nm for PEO6000 dimethyl ether, corresponding to once folded or unfolded extended PEO chains.^52^

Our results show that cyclodextrins are potentially valuable additives to tune polymer crystallization. In the case of PEG (PEO) and likely other crystalline polyethers and related compounds, it can be used to suppress crystallization even at a rather low content of the widely available and inexpensive α-cyclodextrin. Other types of cyclodextrins (e.g., those with different ring sizes or with hydrophobic or other modifications) as well as other rotaxanes are able to thread other classes of polymers and are likely to modulate crystallization behavior, an intriguing subject for further research. Since the enzymatic degradation of polymers is enhanced by reducing polymer crystallinity, cyclodextrin addition, and inclusion complex formation can be used to enhance the biodegradation of polymers.^33,45^

## Supporting Information Available

Experimental methods, DSC data, additional SAXS/WAXS data, tables of studies sample compositions, and SAXS/WAXS data peak indexation (PDF)
