# Supplementary material for: Cyclodextrin-Induced Suppression of the Crystallization of Low-Molar-Mass Poly(ethylene glycol)
Source: ACS Polym Au. 2024 May 2;4(4):266–72. doi: 10.1021/acspolymersau.4c00024 (PMC11328327; doi:10.1021/acspolymersau.4c00024)
Supplement: Supplementary file 1 — lg4c00024_si_001.pdf [file lg4c00024_si_001.pdf]

**Supporting Information**

# Cyclodextrin-Induced Suppression of the Crystallization of Low Molar Mass Poly(ethylene glycol)

Ian W Hamley,[1,*] Valeria Castelletto,[1]

[1] *School of Chemistry, Food Biosciences and Pharmacy, University of Reading, Whiteknights, Reading RG6 6AD, U.K.*

* Author for correspondence. I.W.Hamley@reading.ac.uk

**Table S1**. Composition of samples studied in this work

| **Sample composition wt%** | **Molar ratio [PEG]/[αCD]** |
|---|---|
| 1 wt% PEG1000 | |
| 1 wt% PEG1000 + 0.03 wt% αCD | 33 |
| 1 wt% PEG1000 + 1.6 wt% αCD | 0.7 |
| 1 wt% PEG1000 + 3.6 wt% αCD | 0.3 |
| 1 wt% PEG1000 + 8 wt% αCD | 0.13 |
| 1 wt% PEG1000 + 13 wt% αCD | 0.08[a] |
| 1 wt% PEG3000 | |
| 1 wt% PEG3000 + 0.1 wt% αCD | 3.4 |
| 1 wt% PEG3000 + 5 wt% αCD | 0.07 |
| 1 wt% PEG3000 + 10 wt% αCD | 0.04 |
| 1 wt% PEG6000 | |
| 1 wt% PEG6000 + 0.2 wt% αCD | 0.86 |
| 1 wt% PEG6000 + 5 wt% αCD | 0.04 |
| 1 wt% PEG6000 + 7 wt% αCD | 0.03 |
| 1 wt% PEG6000 + 10 wt% αCD | 0.02 |
| 1 wt% PEG6000 + 13 wt% αCD | 0.01 |

[a] The lower ratio for [PEG1000]/[αCD] is only 0.08 because it is attained at 13 wt% αCD, close to the saturation point of αCD ( αCD solubility in water ≤ 14.5 wt% αCD).

**Table S2.** List of observed and calculated reflections for PEG1000 + 8% αCD (Fig.4)

| **$q_{obs}$/ nm$^{-1}$ ($d_{obs}$, nm)** | **$q_{calc}$/ nm$^{-1}$ ($d_{calc}$, nm)[a]** | **hkl** |
|---|---|---|
| 4.05 (1.55) | 4.17 (1.51) | 001 |
| 5.40 (1.16) | 5.32 (1.18) | 100 |
| 6.74 (0.932) | 6.76 (0.930) | 101 |
| 8.68 (0.724) | 8.98 (0.700) | 110 |
| 9.32 (0.674) | 9.89 (0.635) | 111 |
| 11.53 (0.545) | 11.42 (0.550) | 021 |
| 14.24 (0.441) | 14.38 (0.437) | 211 |
| 14.75 (0.426) | 14.38 (0.437) | 121 |
| 16.12 (0.390) | 16.12 (0.390) | 122 |
| 17.50 (0.359) | 17.50 (0.359) | 014 |

[a] Indexed to a monoclinic (pseudo-hexagonal) unit cell with $a = b = 1.31$ nm, $c = 1.51$ nm, $\gamma^* = 116^o$

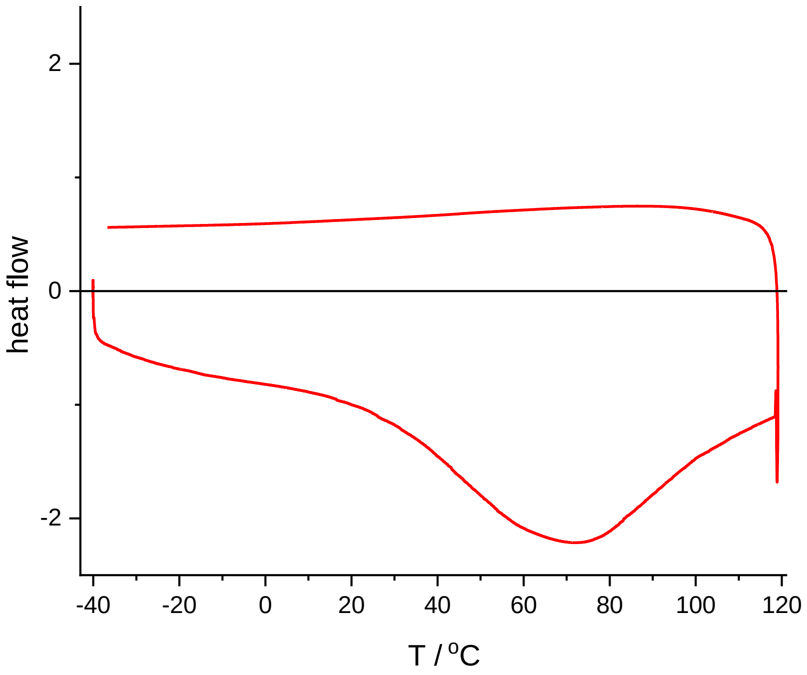


**SI Fig. S1.** DSC data (endo down) measured for αCD. The first heating ramp is from -40 $^o$C to 120 $^o$C and the second cooling ramp from is from 120 $^o$C to -40 $^o$C.

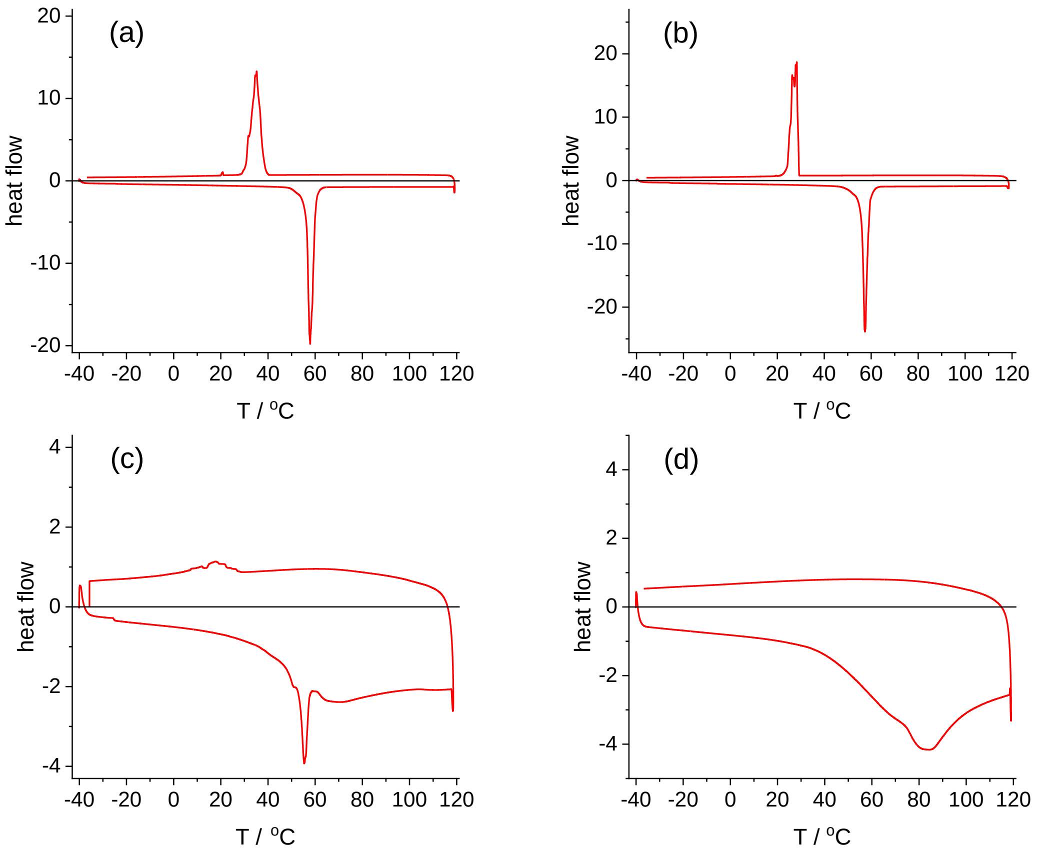


**SI Fig. S2.** DSC data (endo down) measured for PEG3000 with (a) 0, (b) 0.1 wt%, (c) 5 wt%, (d) 10 wt% αCD. The first heating ramp is from -40 °C to 120 °C and the second cooling ramp from is from 120 °C to -40 °C.

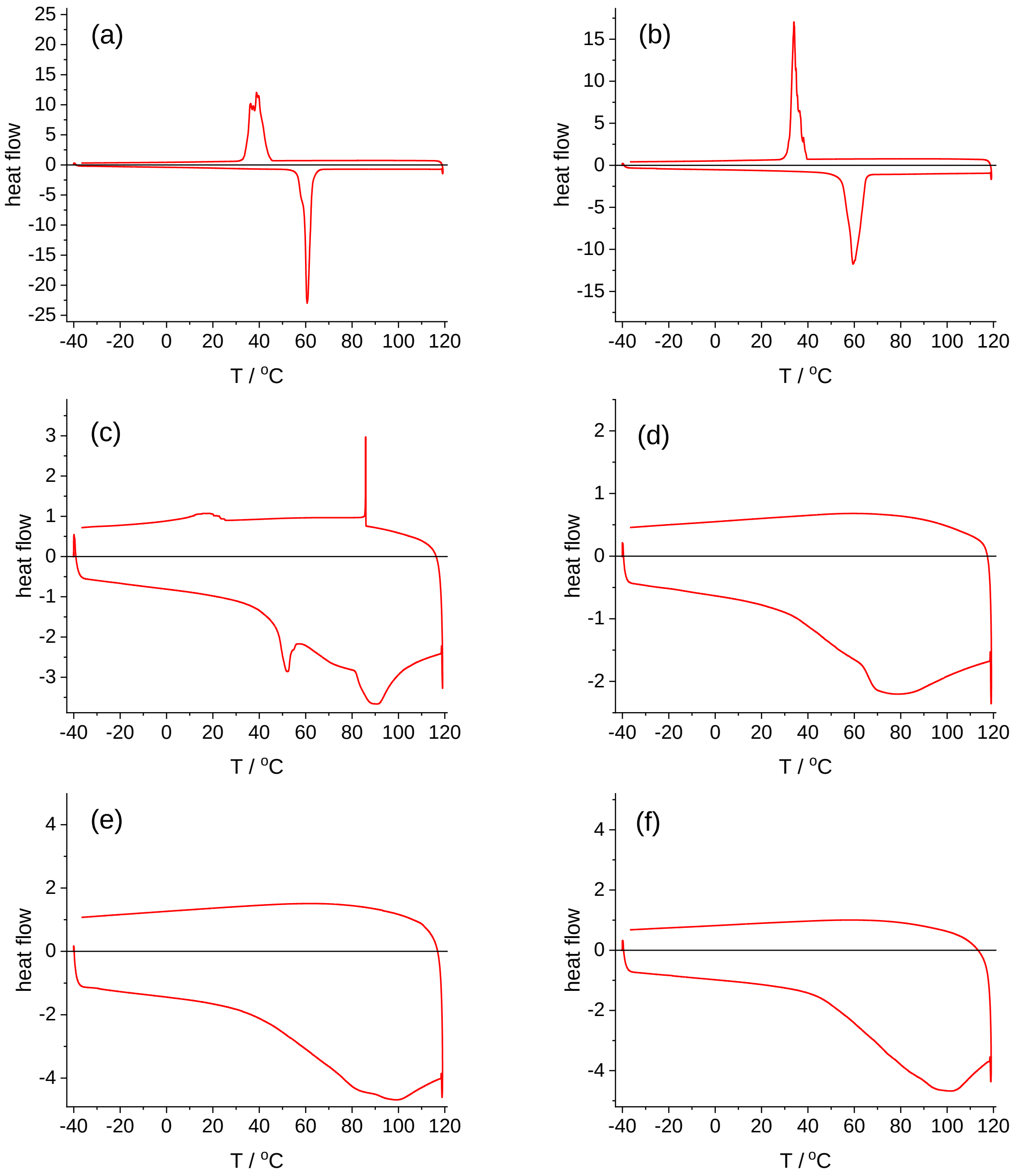


**SI Fig. S3.** DSC data (endo down) measured for PEG6000 with (a) 0, (b) 0.2 wt%, (c) 5 wt%, (d) 7 wt% (e) 10 wt%, (f) 13 wt% αCD. The first heating ramp is from -40 °C to 120 °C and the second cooling ramp from is from 120 °C to -40 °C.

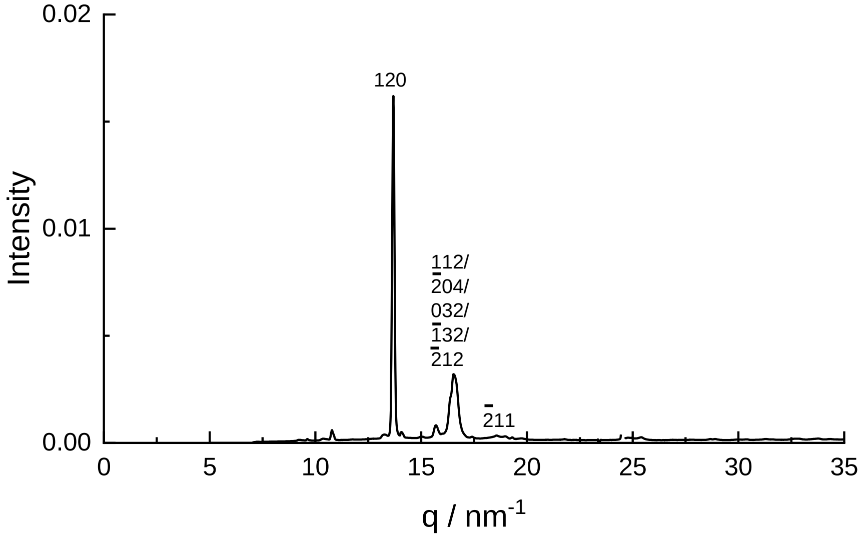


**SI Fig. S4**. WAXS data for PEG1000 at -20 $^{o}$C with selected indexed reflections

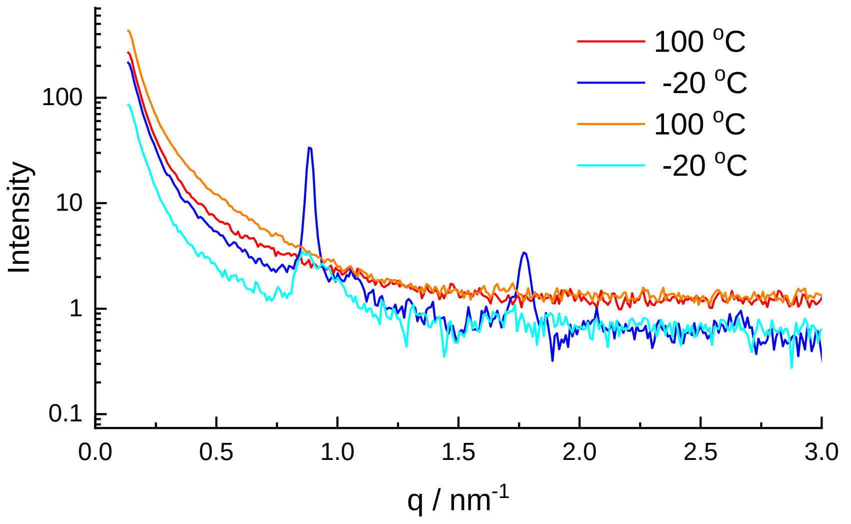


**SI Fig.S5**. Extended *q*-range SAXS data showing higher order peaks due to PEG crystal lamellae (data for PEG1000 + 0.003 wt% αCD, similar results were obtained for PEG1000 alone).

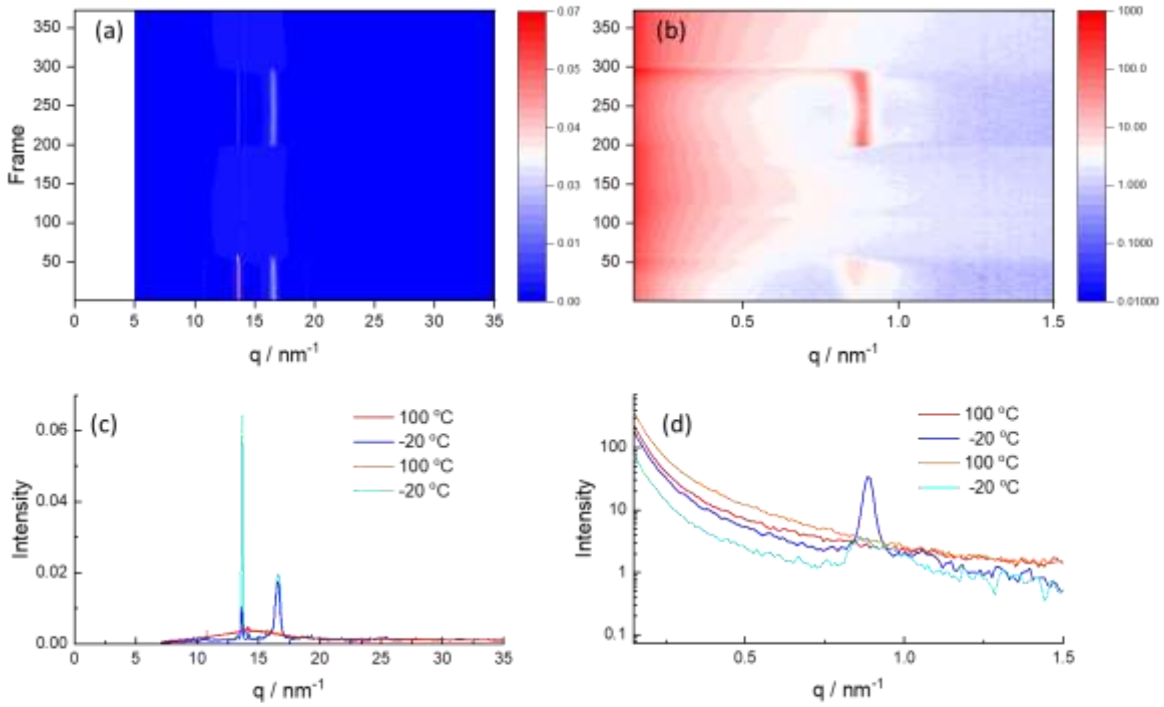


**SI Fig. S6**. SAXS/WAXS data for PEG1000 + 0.003 wt% αCD during a heat/cool/heat cycle at 5 $^{o}$C/min (a) WAXS data heatmap (intensity for each frame stacked vertically), (b) SAXS data heatmap, (c) Selected frames of WAXS data at the temperatures indicated - cyan: -20 $^{o}$C (start), orange: 100 $^{o}$C (first heat), blue -20 $^{o}$C (second cool), red 100 $^{o}$C (second heat), (d) Selected frames of SAXS data (same colour scheme as for WAXS).

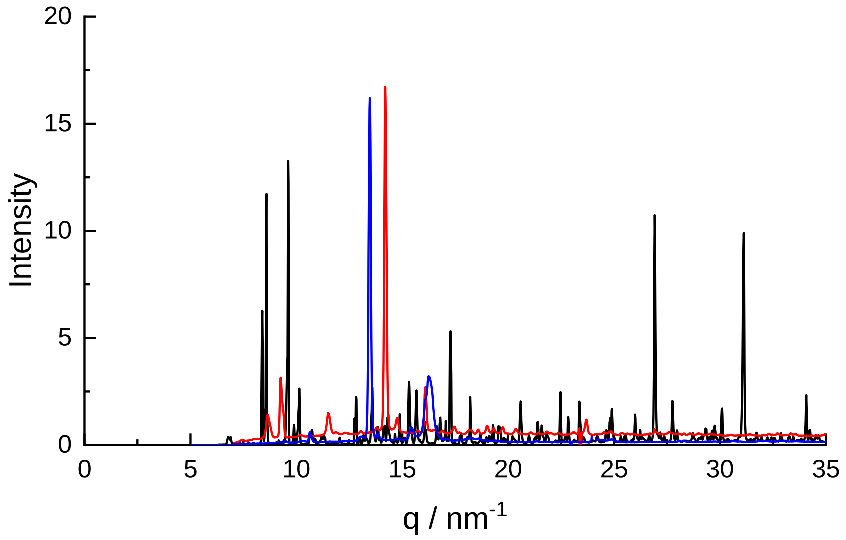


**SI Fig. S7**. WAXS data for PEG1000 + 8wt% αCD at -20 °C (red line) compared to αCD at 22 °C (black line) and PEG1000 at -20 °C (blue line). Data have been scaled for ease of visualization.

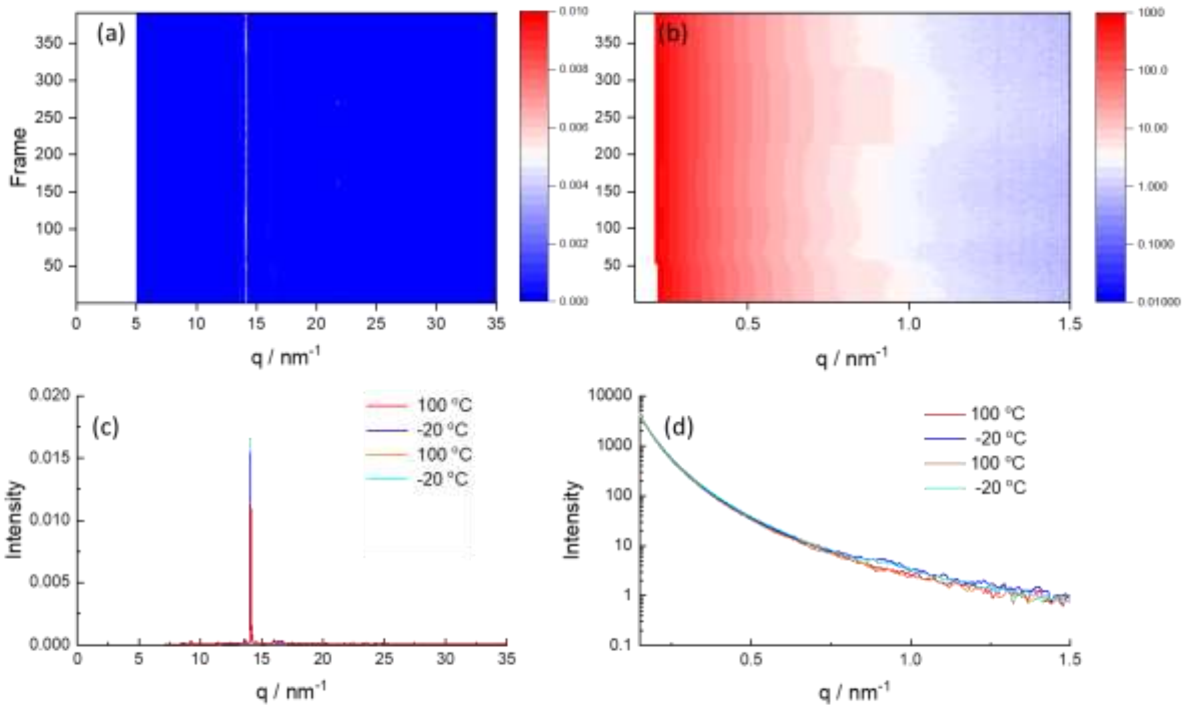


**SI Fig. S8**. SAXS/WAXS data for PEG1000 + 1.6 wt% αCD during a heat/cool/heat cycle at 5 $^{o}$C/min (a) WAXS data heatmap (intensity for each frame stacked vertically), (b) SAXS data heatmap, (c) Selected frames of WAXS data at the temperatures indicated - cyan: -20 $^{o}$C (start), orange: 100 $^{o}$C (first heat), blue -20 $^{o}$C (second cool), red 100 $^{o}$C (second heat), (d) Selected frames of SAXS data (same colour scheme as for WAXS).

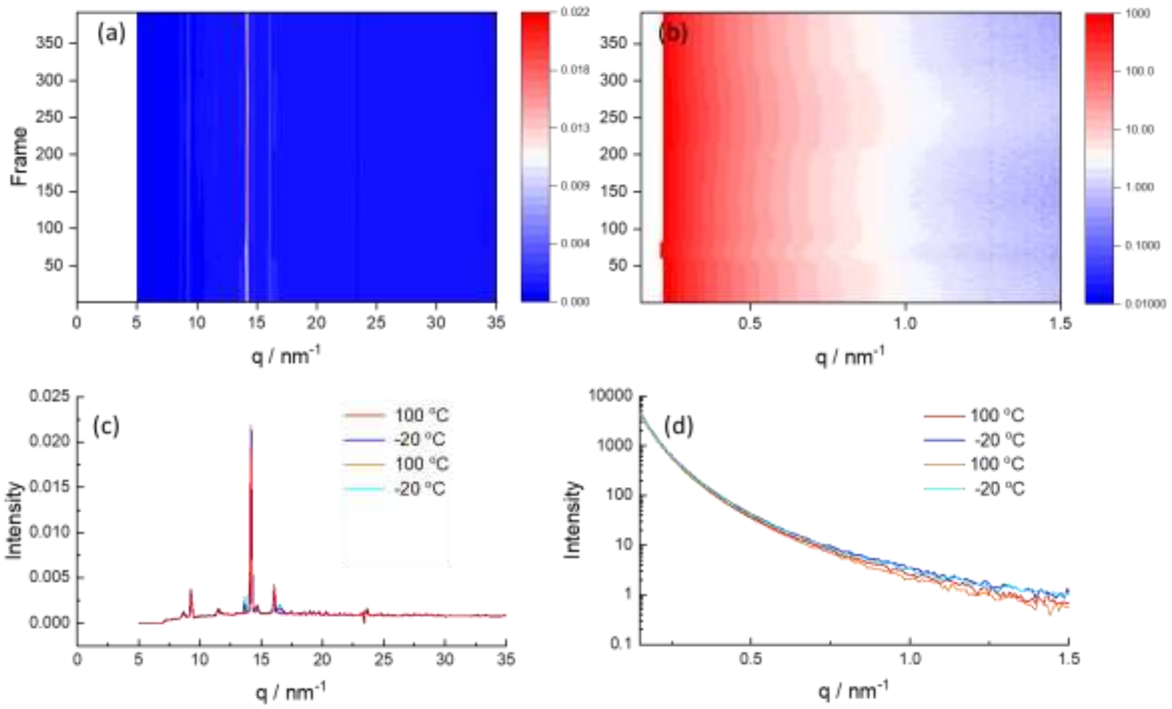


**SI Fig. S9**. SAXS/WAXS data for PEG1000 + 3.6 wt% αCD during a heat/cool/heat cycle at 5 $^{o}$C/min (a) WAXS data heatmap (intensity for each frame stacked vertically), (b) SAXS data heatmap, (c) Selected frames of WAXS data at the temperatures indicated - cyan: -20 $^{o}$C (start), orange: 100 $^{o}$C (first heat), blue -20 $^{o}$C (second cool), red 100 $^{o}$C (second heat), (d) Selected frames of SAXS data (same colour scheme as for WAXS).

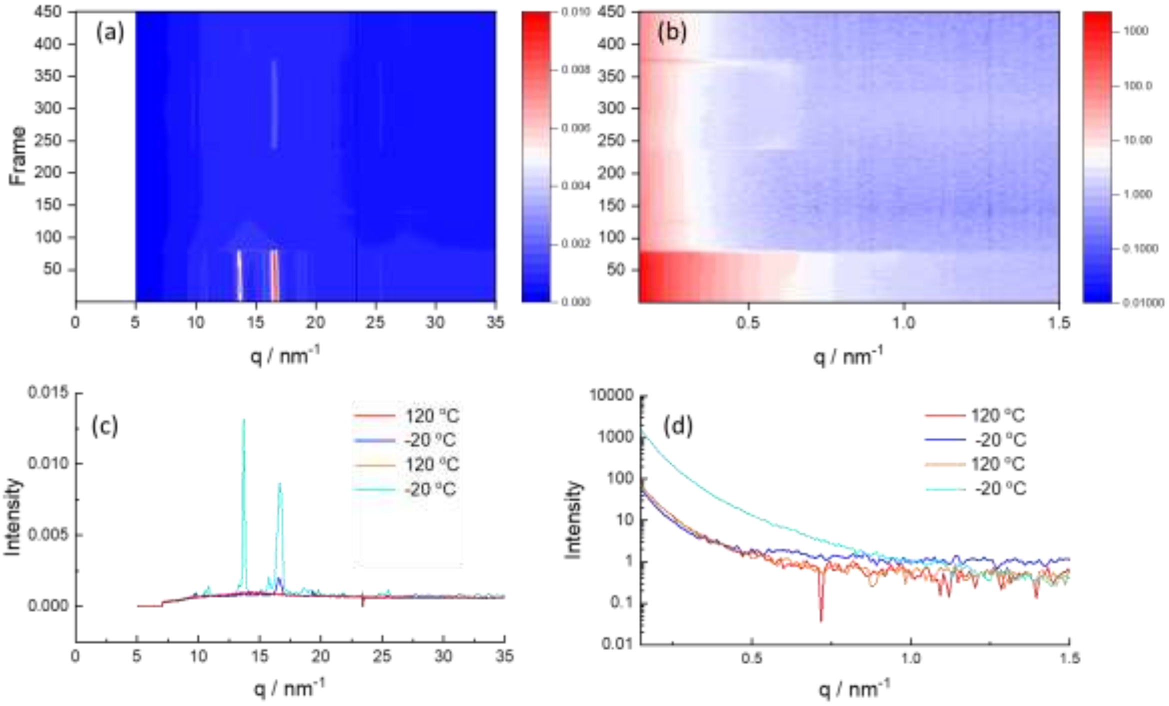


**SI Fig. S10**. SAXS/WAXS data for PEG3000 during a heat/cool/heat cycle at 5 °C/min (a) WAXS data heatmap (intensity for each frame stacked vertically), (b) SAXS data heatmap, (c) Selected frames of WAXS data at the temperatures indicated - cyan: -20 °C (start), orange: 100 °C (first heat), blue -20 °C (second cool), red 100 °C (second heat), (d) Selected frames of SAXS data (same colour scheme as for WAXS).

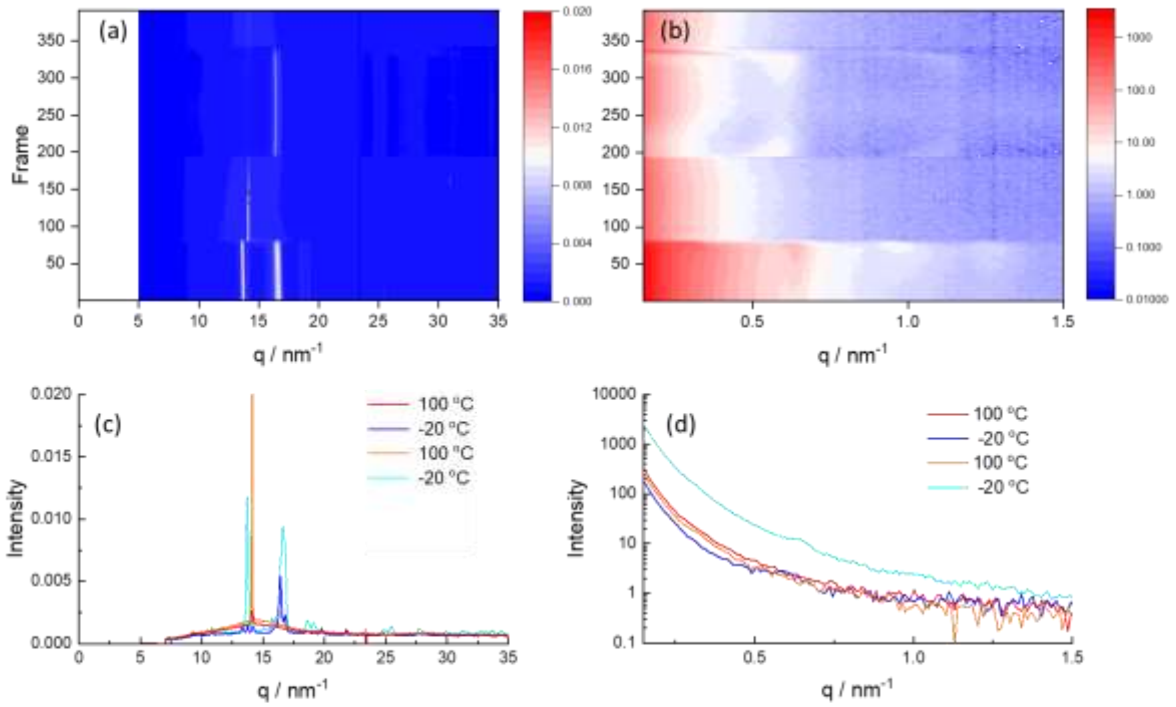


**SI Fig. S11**. SAXS/WAXS data for PEG3000 + 0.1 wt% αCD during a heat/cool/heat cycle at 5 °C/min (a) WAXS data heatmap (intensity for each frame stacked vertically), (b) SAXS data heatmap, (c) Selected frames of WAXS data at the temperatures indicated - cyan: -20 °C (start), orange: 100 °C (first heat), blue -20 °C (second cool), red 100 °C (second heat), (d) Selected frames of SAXS data (same colour scheme as for WAXS).

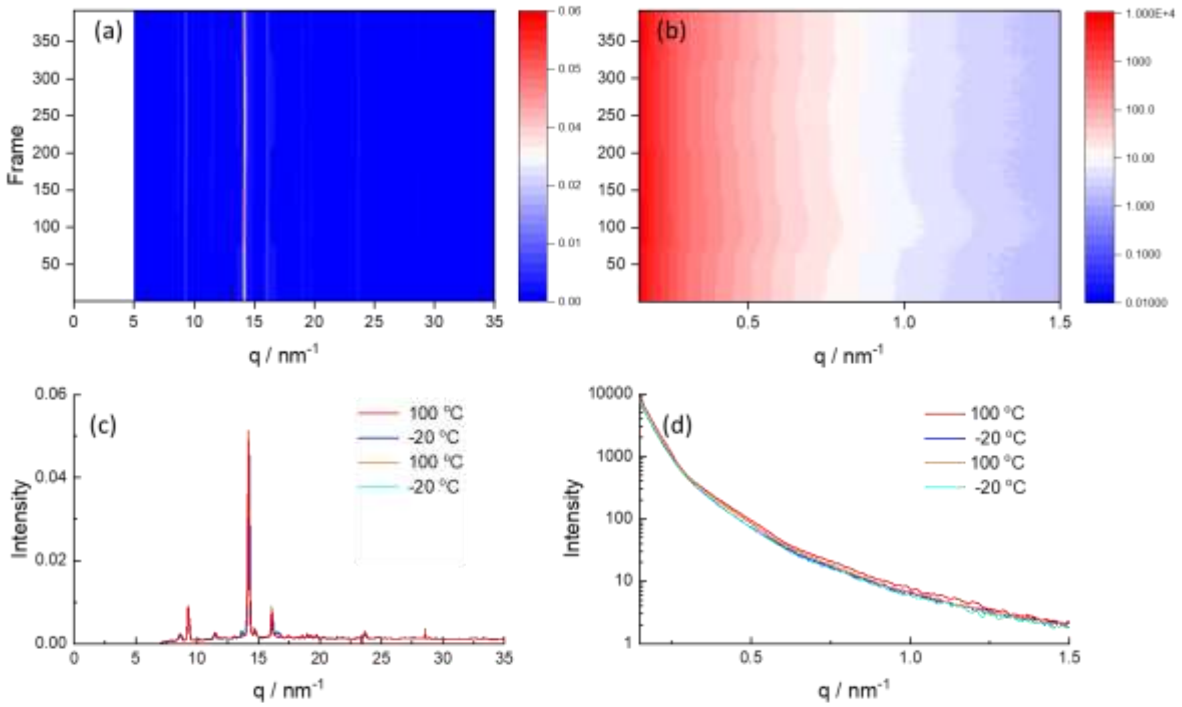


**SI Fig. S12**. SAXS/WAXS data for PEG3000 + 5 wt% αCD during a heat/cool/heat cycle at 5 $^{o}$C/min (a) WAXS data heatmap (intensity for each frame stacked vertically), (b) SAXS data heatmap, (c) Selected frames of WAXS data at the temperatures indicated - cyan: -20 $^{o}$C (start), orange: 100 $^{o}$C (first heat), blue -20 $^{o}$C (second cool), red 100 $^{o}$C (second heat), (d) Selected frames of SAXS data (same colour scheme as for WAXS).

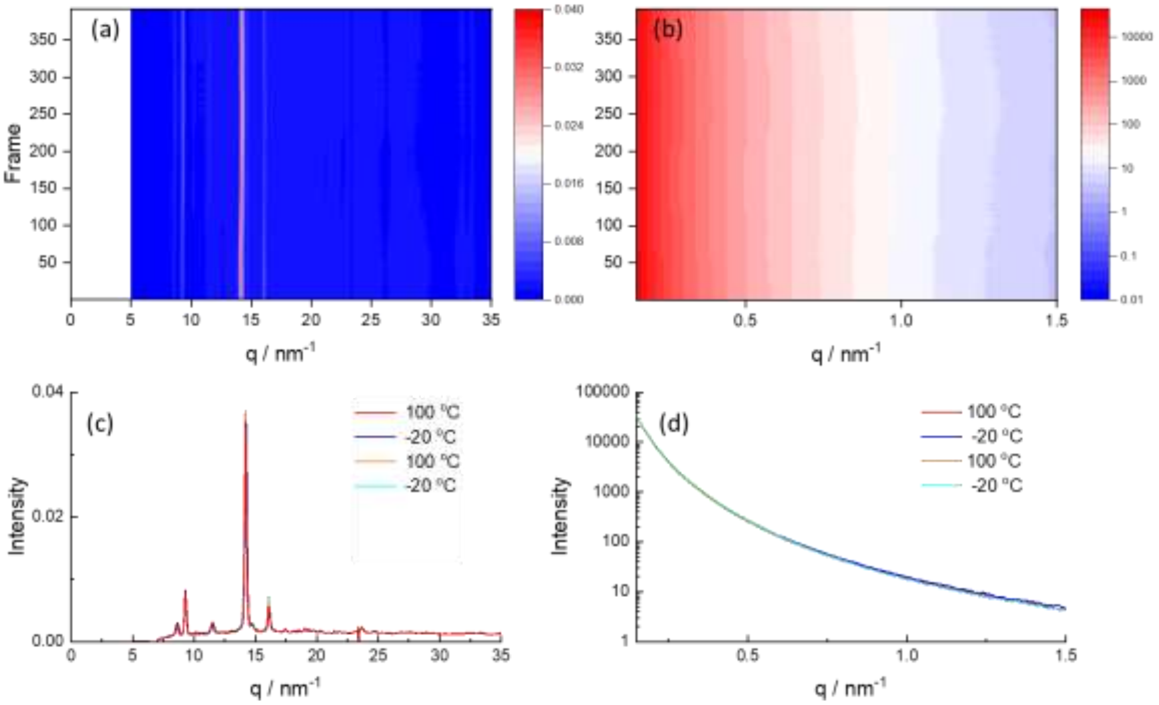


**SI Fig. S13**. SAXS/WAXS data for PEG3000 +10 wt% αCD during a heat/cool/heat cycle at 5 °C/min (a) WAXS data heatmap (intensity for each frame stacked vertically), (b) SAXS data heatmap, (c) Selected frames of WAXS data at the temperatures indicated - cyan: -20 °C (start), orange: 100 °C (first heat), blue -20 °C (second cool), red 100 °C (second heat), (d) Selected frames of SAXS data (same colour scheme as for WAXS).

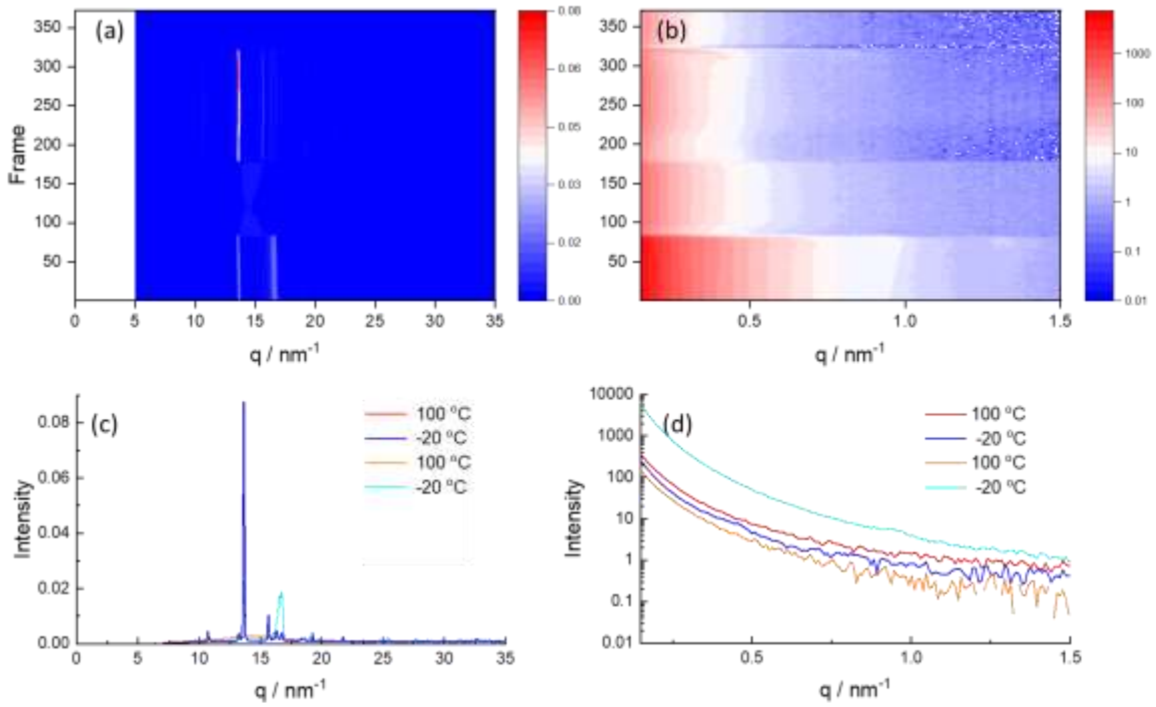


**SI Fig. S14**. SAXS/WAXS data for PEG6000 during a heat/cool/heat cycle at 5 °C/min (a) WAXS data heatmap (intensity for each frame stacked vertically), (b) SAXS data heatmap, (c) Selected frames of WAXS data at the temperatures indicated - cyan: -20 °C (start), orange: 100 °C (first heat), blue -20 °C (second cool), red 100 °C (second heat), (d) Selected frames of SAXS data (same colour scheme as for WAXS).

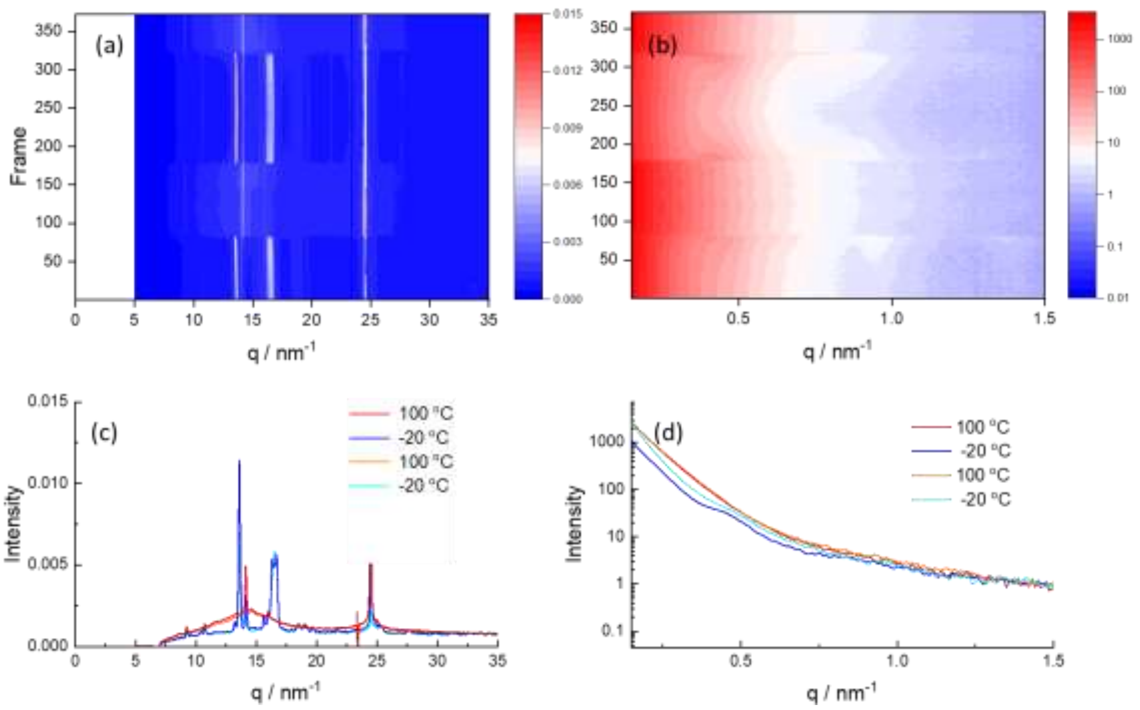


**SI Fig. S15**. SAXS/WAXS data for PEG6000 + 0.2 wt% αCD during a heat/cool/heat cycle at 5 °C/min (a) WAXS data heatmap (intensity for each frame stacked vertically), (b) SAXS data heatmap, (c) Selected frames of WAXS data at the temperatures indicated - cyan: -20 °C (start), orange: 100 °C (first heat), blue -20 °C (second cool), red 100 °C (second heat), (d) Selected frames of SAXS data (same colour scheme as for WAXS).

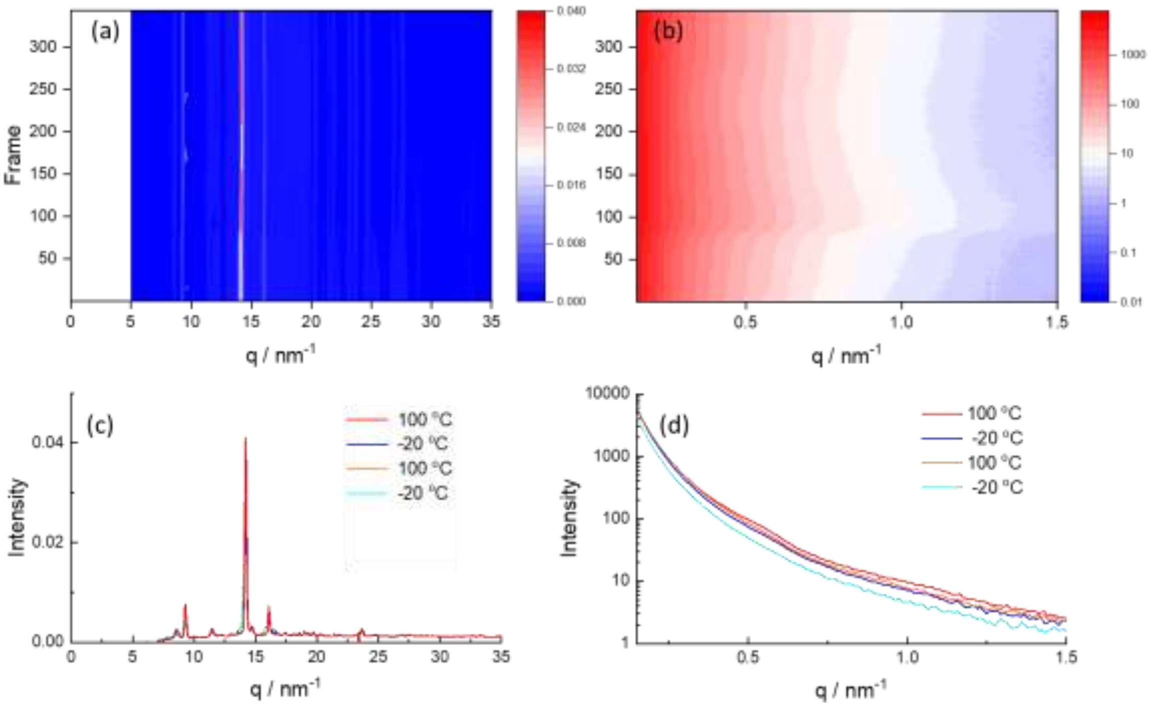


**SI Fig. S16**. SAXS/WAXS data for PEG6000 + 5 wt% $\alpha$CD during a heat/cool/heat cycle at 5 $^{o}$C/min (a) WAXS data heatmap (intensity for each frame stacked vertically), (b) SAXS data heatmap, (c) Selected frames of WAXS data at the temperatures indicated - cyan: -20 $^{o}$C (start), orange: 100 $^{o}$C (first heat), blue -20 $^{o}$C (second cool), red 100 $^{o}$C (second heat), (d) Selected frames of SAXS data (same colour scheme as for WAXS).

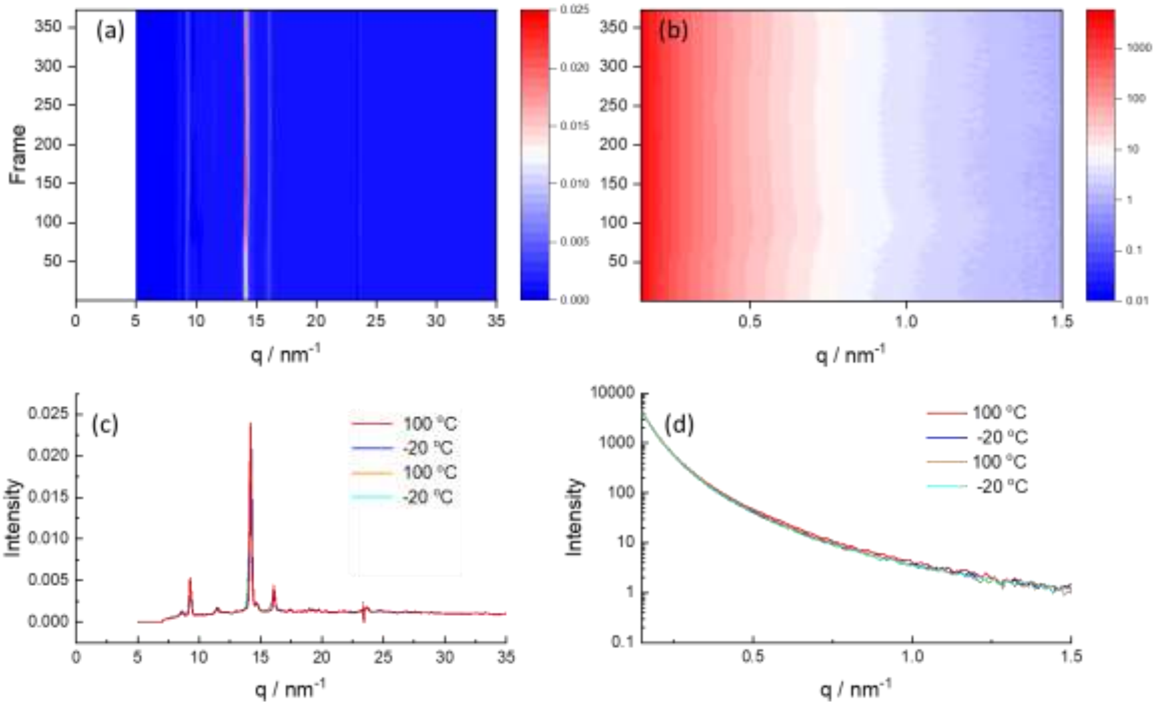


**SI Fig. S17**. SAXS/WAXS data for PEG6000 + 7 wt% αCD during a heat/cool/heat cycle at 5 $^{o}$C/min (a) WAXS data heatmap (intensity for each frame stacked vertically), (b) SAXS data heatmap, (c) Selected frames of WAXS data at the temperatures indicated - cyan: -20 $^{o}$C (start), orange: 100 $^{o}$C (first heat), blue -20 $^{o}$C (second cool), red 100 $^{o}$C (second heat), (d) Selected frames of SAXS data (same colour scheme as for WAXS).

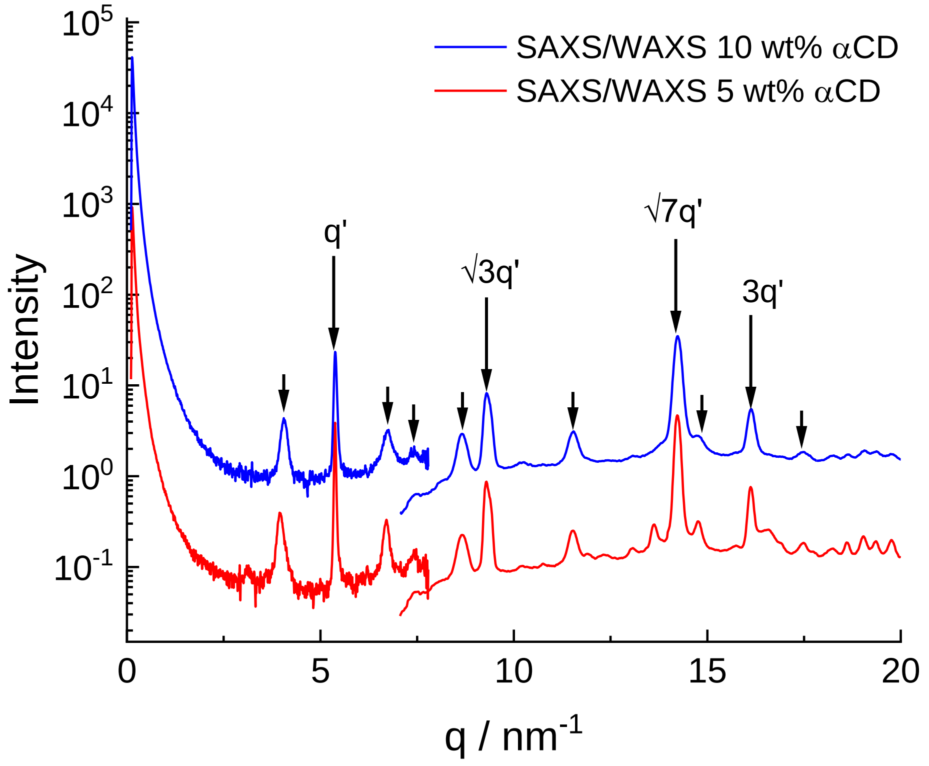


**SI Fig.S18**. Combined SAXS/WAXS data (at -20 ºC, first cooling) for PEG3000 mixtures with αCD content as indicated. The WAXS data intensity has been scaled to be at approximately the same level of that of the SAXS data, and data is offset for ease of visualization. Peaks due to PEG/αCD complex formation are indicated in black, with the main hexagonal lattice peaks indexed with q′ notation.

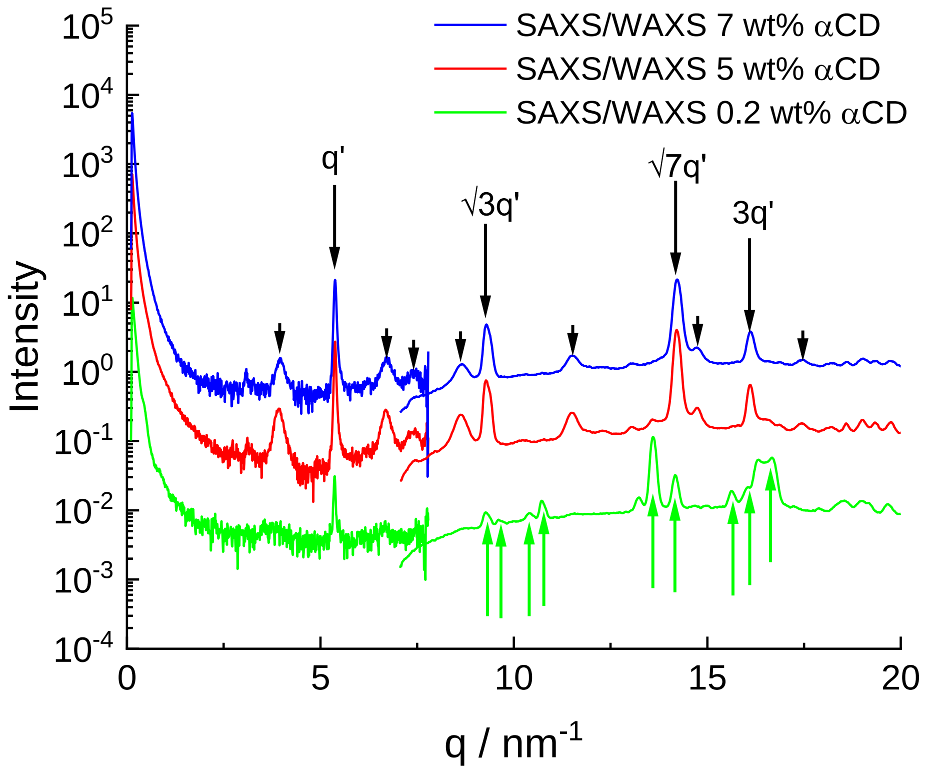


**SI Fig.S19**. Combined SAXS/WAXS data (at -20 ºC, first cooling) for PEG6000 mixtures with αCD content as indicated. The WAXS data intensity has been scaled to be at approximately the same level of that of the SAXS data, and data is offset for ease of visualization. Peaks due to PEG/αCD complex formation are indicated in black, with the main hexagonal lattice peaks indexed with q′ notation. Peaks due to PEG crystallization are highlighted with green arrows.

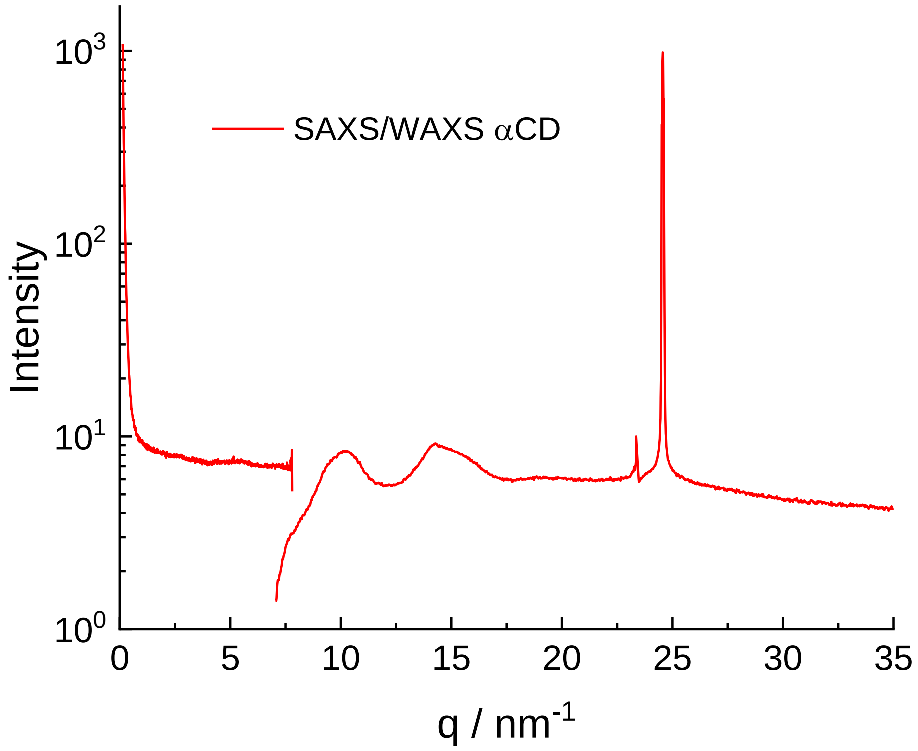


**SI Fig.S20**. Combined SAXS/WAXS data (at 20 °C) for αCD. The WAXS data intensity has been scaled to be at approximately the same level of that of the SAXS data.
